# Elevated serum complement factors 3 and 4 are strong inflammatory markers of the metabolic syndrome development: a longitudinal cohort study

**DOI:** 10.1038/srep18713

**Published:** 2016-01-04

**Authors:** Zhenfang Liu, Qin Tang, Jing Wen, Yan Tang, DaMin Huang, Yuzhen Huang, Jinling Xie, Yawen Luo, Min Liang, Chunlei Wu, Zheng Lu, Aihua Tan, Yong Gao, Qiuyan Wang, Yonghua Jiang, Ziting Yao, Xinggu Lin, Haiying Zhang, Zengnan Mo, Xiaobo Yang

**Affiliations:** 1 Center for Genomic and Personalized Medicine, Guangxi Medical University, Nanning, Guangxi 530021, China; 2 Guangxi Key Laboratory of genomic and personalized medicine, Nanning, Guangxi 530021, China; 3 Guangxi Collaborative Innovation Center for genomic and personalized medicine, Nanning, Guangxi 530021, China; 4 Department of Occupational Health and Environmental Health, School of Public Health, Guangxi Medical University, Nanning; 5 Department of Endocrinology, First Affiliated Hospital ofGuangxi Medical University; 6 Department of Urology, First Affiliated Hospital of Xinxiang Medical College, Xinxiang, Henan Province, China; 7 Institute of Urology and Nephrology, First Affiliated Hospital of Guangxi Medical University, Nanning, Guangxi530021, China; 8 Department of chemotherapy, The Affiliated Tumor Hospital of Guangxi Medical University, Nanning, Guangxi 530021, China

## Abstract

An epidemiological design, consisting of cross-sectional (n = 2376) and cohort (n = 976) studies, was adopted to investigate the association between complement factors 3 (C3) and 4, and the metabolic syndrome (MetS) development. In the cross-sectional study, the C3 and C4 concentrations in the MetS group were higher than those in the non-MetS group (all *P* < 0.001), and the levels of immune globulin M (IgM), IgA, IgE, and IgG exhibited no significant differences between MetS and non-MetS (all *P* > 0.050). After multi-factor adjustment, the odds ratios (ORs) in the highest quartile of C3 and C4 concentrations were 7.047 (4.664, 10.648) and 1.961 (1.349, 2.849), respectively, both *P*_trend_ < 0.050. After a 4 years follow-up, total 166 subjects were diagnosed with MetS, and the complement baseline levels from 2009 were used to predict the MetS risk in 2013. In the adjusted model, the relative risks (RRs) in the highest quartile of C3 and C4 levels were 4.779 (2.854, 8.003) and 2.590 (1.567, 4.280), respectively, both *P*_trend_ < 0.001. Activation of complement factors may be an important part of inflammatory processes, and our results indicated that the elevated C3 and C4 levels were independent risk factors for MetS development.

Metabolic syndrome (MetS) affects approximately 20%–25% of the adult population worldwide^1^; this multicomponent abnormality includes hyperglycemia, dyslipidemia, abdominal obesity, and hypertension^2^. MetS has attracted considerable interest as a risk factor for the development of type 2 diabetes mellitus^3^ and cancers, such as colorectal cancer^4^. A prospective population study identified that men with MetS have a 1.5-fold risk of developing coronary heart disease^5^.

Systemic low-grade inflammation is responsible for immune function activation and contributes to metabolic disorders^6^. Elevated immune globulin A (IgA), IgE, and IgG, but not IgM, are related to myocardial infarction and cardiac death in males with hyperlipidemia^7^. High-sensitivity C-reactive protein is an acute-phase response marker and a well-known independent predictor of metabolic dysfunction, such as cardiovascular disease^8^, hypertensi^9^,^10^, and diabetes^11^,^12^. Similarly, elevated levels of other acute-phase reactants, such as interleukin-6^13^, and proinflammatory cytokines, such as tumor necrosis factor-α^14^ and fibrinogen^15^, can also be risk markers of metabolic diseases. Moreover, insulin resistance (IR)^16^,^17^, obesity^18^, and hypercholesterolemia^19^ as components of MetS are usually independently interrelated to an increase in inflammatory markers. Given these findings, low-grade inflammation exerts significant effects on the development of metabolic dysfunction.

The complement system response to inflammation and infection is significant in innate and adaptive immune mechanisms. C3 and C4, mainly produced by the liver^20^, are the major plasma proteins of the immune system complement pathways^21^. Common genetic variants at the C3 locus have been suggested to be associated with risk of MetS and its components^22^. A cohort study of 1220 adults from a general population proved that the fasting level of C3 was strongly associated with abdominal obesity and blood pressure^23^. C3 is also associated with postprandial triglyceride metabolism, higher homeostasis model assessment of IR (HOMA-IR), and diabetes development^24^,^25^,^26^. Furthermore, elevated C3 concentrations show greater predictive value than insulin or C reactive protein for the incidence of fitness and fatness^27^. Adipose tissue may be considered as an immunological organ^28^, and adipose tissue inflammation of obese subjects contributes to the development of IR and metabolic dysfunction^29^. High expressions of C3 and C4 are related to adipose tissue variables and involved in the development of visceral adiposity^30^.

Therefore, the complement system response to inflammation and infection may play an important role in MetS expression. However, to our knowledge, large-scale epidemiological studies that demonstrate the association between C3 and C4, and MetS morbidity are limited. In the present investigation, we conducted a cross-sectional and longitudinal population-based study to elucidate whether C3, C4, or immune globulin concentrations are associated with MetS risk, or predict MetS incidence.

## Results

### Participants’ selection of the study

**Figure 1:**
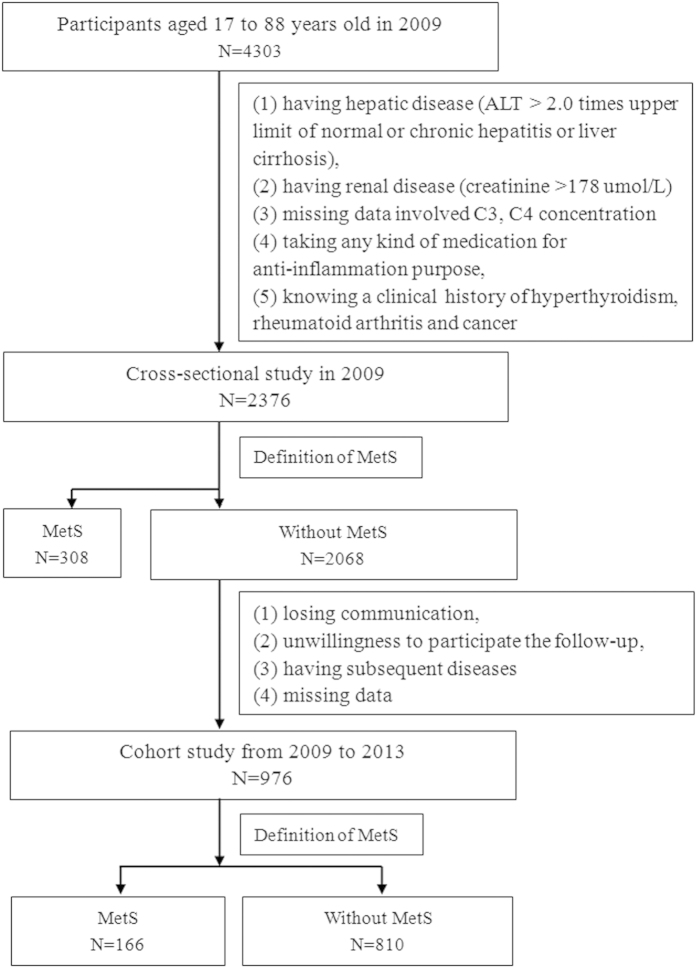
Flow chart for selection of study participants. Based on the inclusion criteria, 2376 males participated in the cross-sectional study and 976 males participated in the cohort study.

Figure 1 shows the procedures for selecting participants in the study (n = 4303). Among the 4303 participants aged 17–88 years old, 2376 eligible male participants were selected in the cross-sectional study based on the exclusion criteria. A total of 308 (12.96%) and 2068 subjects diagnosed with MetS and without MetS (non-MetS) were identified, respectively, based on the definition of MetS. After applying the exclusion criteria during the follow-up period, 976 participants were included in the cohort study from the 2068 participants without MetS from 2009 to 2013. After a 4 year follow-up, 166 subjects (17.01%) were diagnosed with MetS.

### General characteristics investigation

Table 1 shows the general characteristics of male participants. The C3 and C4 levels of participants with MetS were 15% higher than those without MetS (both *P* < 0.001). However, no significant differences were observed in IgM (*P* = 0.148), IgA (*P* = 0.115), IgE (*P* = 0.699), and IgG (*P* = 0.269) between the two subjects groups. The smoking status between MetS and non-MetS participants was also not significantly different (*P* = 0.070).

### Correlation coefficients between C3 and C4 concentrations and metabolic variables

Pearson’s correlation coefficient showed the relationship between the C3 and C4 levels and the risk factors of MetS after adjusting the confounding factors (Table 2). C3 levels were significantly positively correlated with body mass index (BMI), waist circumference (WC), systolic blood pressure (SBP), diastolic blood pressure (DBP), triglyceride (TRIG), glucose (GLU), and decreased high-density lipoprotein (HDL) (all *P* < 0.001). C4 levels were also related to the risk parameters except for TRIG (*P* = 0.153) and GLU (*P* = 0.057). In addition, C3 levels were highly correlated with BMI and WC (both R = 0.436), whereas C4 levels were strongly correlated with BMI (R = 0.221). No significant association among IgE, IgG, IgM, IgA, and the components of MetS was found after adjustment of multiple factors (Supplemental Table 1 online).

### Association between C3 and C4 levels and MetS in the cross-sectional study

Table 3 presents the association between the concentration quartiles of C3 and C4 and MetS by logistic regression analysis. In the unadjusted model (model 1), the odds radio (ORs) of MetS in the highest quartile of C3 and C4 levels were 6.240 and 2.213, respectively (both *P* < 0.001). After multi-factor adjustment (model 2), the ORs in the highest quartile of C3 and C4 concentrations were 7.047 (*P*_trend_ < 0.001) and 1.961(*P*_trend_ = 0.041). Nevertheless, binary logistic regression analysis showed that the ORs and 95% confidence intervals (CIs) of MetS for IgE, IgG, IgM, and IgA were mostly non-significant (Supplemental Table 2 online).

### C3 and C4 levels predict MetS incidence in the cohort from 2009 to 2013

**Figure 2:**
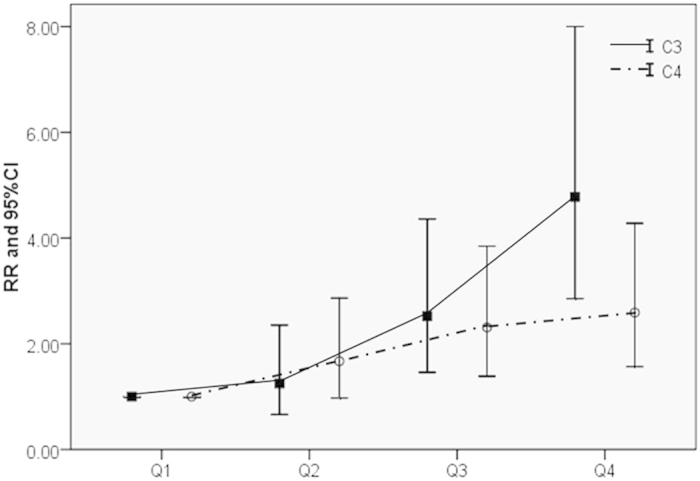
RRs of MetS presence in 2013 according to the quartiles of C3 and C4 baseline levels from 2009. RRs exhibited an increasing trend when the quartiles of C3 and C4 increased (*p* < 0.001).

Relative risks (RRs) for MetS development and the quartiles of C3 and C4 concentrations are shown in the final Cox regression analysis table (Table 4). In unadjusted analysis (model 1), the RRs of MetS for the highest quartile of C3 and C4 concentrations were 4.556 and 2.711, respectively (both *P* < 0.001). After multi-factor adjustment (model 2), the highest C3 concentrations were associated with 4.7 times higher risk of developing MetS compared with their counterparts. Data showed that the incidence of MetS increased with rising quartiles (*P*_trend_ < 0.001) (Fig. 2). Similarly, the chance of developing MetS was 2.590 times higher in individuals with the highest quartile of C4 concentrations compared with those with the lowest quartile of C4 concentrations (*P* < 0.001).

## Discussion

Both C3 and C4, but not IgE, IgG, IgM, and IgA, were related to independent components of MetS, and elevated C3 or C4 was positively associated with MetS in the cross-sectional study. We also found that the high baseline C3 and C4 levels indicated increased risk of MetS over a 4-year follow-up study.

Chronic inflammation is associated with MetS^6^. Meanwhile, the activation of complement system is an important part of inflammatory processes that interferes with normal metabolism and insulin signaling^17^. In the present study, baseline C3 and C4 levels, but not IgM, IgA, IgE, and IgG levels, showed significant differences between MetS and non-MetS groups. A previous cross-sectional study reported that a borderline relationship exists between IgM levels and MetS. This finding may be mediated by lipid metabolism disorder in males (*P* = 0.07) when IgM levels are significantly related to elevated TRIG and decreased HDL^31^. However, in the present study, the IgM, IgA, IgE, and IgG exhibited no significant effects on MetS and its components among the subjects (Supplemental Tables 1 and 2 online). Therefore, we propose that the activation of inflammatory pathways, particularly the complement pathway, mainly affects MetS incidence.

Several investigations, mainly case–control or cross-sectional studies reported consistent results. For example, a case–control study found that C3 concentrations are significantly related to serum TRIG, GLU, and BP levels^32^. Individuals with elevated C3 concentrations exhibit three-fold higher risk of MetS compared with those in the bottom 50th percentile, and higher MetS risk among high dietary fat consumers and smokers^33^. A cross-sectional study of Prospective Investigation of the Vasculature in Uppsala Seniors study also demonstrated that C3 and C4 are significantly related to the occurrence of MetS in 1016 older people (≥70 years old)^29^. By contrast, the current study has a larger sample size and relatively young population, with an average age of 42.6 years old. In a cross-sectional population-based survey of Wamba *et al.*^34^, they proposed that acylation-stimulating protein, but not complement C3, is associated with MetS components in 1603 Chinese children and adolescents (6–18 years old). In another study of Wamba *et al.* on children aged 2–6 years old, they reported a small but significant increase in plasma C3 in young obese subjects^35^. Thus, these results indicate that C3 and C4 may affect MetS prevalence only in adult population, but not significantly in children.

A cohort study focused on cardiometabolic risk and MetS; by analyzing the association between C3 and the incidence of MetS in 265 women and 230 men, the study reported that C3 predicted the risk of MetS in women but not in men^23^. However, basing on the results from the present cohort study, we propose that elevated C3 is an independent risk factor for the development of MetS in a large sample of men. This relationship still existed in men, even after adjustment for other confounding factors. Moreover, the present study found that increased C4 is another potential factor in predicting MetS.

The pathogenesis of MetS caused by activation of complement system is not clearly elucidated, and several pathways may explain how complement factors affect MetS. C3 and C4, as the major plasma proteins of the complement pathway, play a crucial role in the immune system^21^. High levels of C3 may cause high C3a and C5a, these anaphylatoxins mediate inflammatory processes by acting on their respective receptors (C3aR and C5aR)^36^,^37^. *In vivo*, studies showed that C3aR and C5aR knock-out mice present reduced macrophage infiltration and improved insulin sensitivity^32^,^37^,^38^. C3a and C5a function as hormones that have insulin-like effects on 3T3-L1 adipocytes through increasing glucose and fatty acid uptake while inhibiting cAMP transmission and lipolysis to promote energy conservation^39^. In addition, C3a-desArg is a degraded product of C3 and can stimulate lipogenesis in adipose cells and triglyceride synthesis^40^,^41^, and also promote islets secreting glucose-dependent insulin^38^,^42^. The metabolism of triglyceride was accelerated with increasing C3a-desArg levels. This phenomenon increased the plasma triglyceride levels, thereby explaining the IR in obese people and MetS^29^. Zhou *et al.* suggested that C3 was involved in mediating epithelial-to-mesenchymal transition and in activating intrarenal RA systems with the influence of renin generation. The finding indicated that high level C3 could increase intrarenal Ang II concentration and BP levels^43^, which could explain the association between C3 and hypertension.

The present work is the first to combine cross-sectional and longitudinal studies to investigate the casual relationship between complement activation and MetS. The strengths of our study include population-based sample, large sample size, and relatively high response rate, which may improve the credibility of the results.

Nevertheless, several limitations should be highlighted. First, this study was conducted only among adult males. The results may lack sufficient evidence to indicate the relationships in children and females. Second, no diet intake data or detailed lifestyles were investigated. Therefore, the effects of diet on MetS were not analyzed in our study.

In conclusion, activation of complement factors, as an important part of inflammatory processes, may directly affect MetS incidence. Elevated C3 and C4 levels were independent risk factors for the development of MetS in Chinese male population. Further studies are urgently needed to explore the molecular mechanisms of activation of complement factors acting on MetS.

## Materials and Methods

### Study population

This study is part of Fangchenggang Area Males Health and Examination Survey (FAMHES). FAMHES is a population-based epidemiological cohort study performed in Guangxi, China^44^. A total of 4303 eligible Chinese men aged 17 to 88 years completed face-to-face interview and physical examination from September 2009 to December 2009 at the Medical Centre of Fangchenggang First People’s Hospital.

#### Ethics

This study was approved by the Ethics and Human Subject Committee of Guangxi Medical University and was conducted in accordance with the Declaration of Helsinki. All participants were given and signed a written informed consent form prior to the study.

### Cross-sectional study

The exclusion criteria are as follows:^1^ presence of hepatic disease (ALT > 2.0 times upper limit of normal or chronic hepatitis or liver cirrhosis), 2presence of renal disease (creatinine >178 μmol/l), 3missing data on C3 and C4 concentrations, 4taking anti-inflammatory medication, and^5^ clinical history of hyperthyroidism, rheumatoid arthritis, and cancer. Participants who had signs of high fasting blood glucose levels and high BP at the initial assessment were included in the study. Among the 4303 participants, 2376 with complete data were included in the cross-sectional study in 2009.

### Cohort study

A total of 2376 participants without MetS at baseline in 2009 were followed up for 4 years. The participants completed the same face-to-face interview and physical examination in 2013. We excluded members who had one or more of the following criteria:^1^ loss to follow-up, 2unwillingness to participate, 3subsequent diseases that are unsuitable for participation, and^4^ missing data on anthropometric measurements or clinical biochemistry assays in 2013. A total of 976 men participated in the follow-up exam.

### Data collection

We collected the data from Fangchenggang First People’s Hospital Medical Examination Center. Participants’ age, smoking habits, alcohol consumption, and health status were obtained through a face-to-face interview. Subjects who had ever smoked cigarettes for six months or longer were classified as smokers, and others were considered as non-smokers. Drinkers were defined according to alcohol consumption once or more a week, and others were considered as non-drinkers. Subjects who had exercised two hours or more each week were classified as regular physical activity, and otherwise subjects were classified as irregular physical activity^45^. Participants were considered to have a family history of chronic diseases if one of the following is present in participants’ parents or siblings: hypertension, type 2 diabetes mellitus, coronary heart disease, and stroke^46^.

Anthropometric measurements were conducted by trained personnel using a standard protocol. Standing heights were measured using a vertical telescopic stadiometer with a horizon headboard on the top to the nearest 0.1 cm. Participants were weighed without shoes to the nearest 0.1 kg. BMI was calculated as weight divided by height squared (kg m^−2^). WC was assessed midway between the lower rib margin and the iliac. BP was measured twice on each visit after 5 min of rest with an oscillometric precision blood pressure instrument on the right arm by trained nurses.

### Laboratory measurements

Overnight fasting venous blood samples were obtained between 8 a.m. and 10 a.m. The samples were transported on ice in an upward position to the testing center of the Department of Clinical Laboratory at the First Affiliated Hospital of Guangxi Medical University in Nanning within 2 h. Sera were separated using a centrifuge within 15–25 min and stored at −80 °C until analysis. Blood lipids and serum glucose were determined using the enzymatic assay on a Dimension-RxL chemistry analyzer (Dade Behring, USA). IgE was measured by electro-chemiluminescence immunoassay on COBAS 6000 system E601 (Elecsys module) immunoassay analyzers (Roche Diagnostics, IN, Germany) with the same batch of reagents. C3, C4, IgG, IgA, and IgM were measured with a Hitachi 7600 autoanalyzer (Hitachi Corp., Japan) in the testing center of the First Affiliated Hospital of Guangxi Medical University. The coefficients of variation were 2.16% for C3, 2.51% for C4, 4.67% for IgM, 4.97% for IgG, 3.57% for IgA, and 3.3% for IgE.

### Definition of MetS cases

Based on the National Cholesterol Education Program – Adult Treatment Panel III definition as modified by the American Heart Association/National Heart, Lung and Blood Institute^41^, individuals with MetS were identified having arbitrarily three or more of the following criteria: (1) WC ≥ 90 cm, (2) TRIG ≥ 1.7 mmol/l, (3) HDL < 1.03 mmol/l, (4) SBP ≥ 130 mmHg/DBP ≥ 85 mmHg or current use of hypotensive drugs or have a history of hypertension, and (5) fasting GLU ≥ 5.6 mmol/l or previously diagnosed with type 2 diabetes mellitus or taking medications to control hyperglycemia.

### Statistical methods

Continuous variables were described as mean and standard deviation (SD) ranges with normal distribution, median, and 25–75 percentile with abnormal distribution, and categorical variables were described as proportions. Two-sided *t* test and Mann–Whitney U test were used to analyze the differences of variables between the MetS and normal groups. Pearson’s coefficient was employed to describe the correlations of C3 and C4 with metabolic variables after multi-factor adjustment (including age, smoking, drinking status, physical activity, and family history of chronic diseases). Estimates of the likelihood of MetS and 95% CIs were obtained using logistic regression analysis in models with or without adjustment of multi-factors. After the 4-year follow-up, Cox proportional hazard regression was conducted to compute RRs and 95% CI for MetS after adjustment or without adjustment for multi-factors. A linear trend across increasing quartiles was tested with a mean value of each quartile as an ordinal variable. All statistical analyses were performed using SPSS (Chicago, IL, USA) for Windows 16.0. Statistical tests were two-sided, and *P* < 0.05 was considered statistically significant.

## Additional Information

**How to cite this article**: Liu, Z. *et al.* Elevated serum complement factors 3 and 4 are strong inflammatory markers of the metabolic syndrome development: a longitudinal cohort study. *Sci. Rep.* **6**, 18713; doi: 10.1038/srep18713 (2016).

## Supplementary Material

Supplementary Information

## Figures and Tables

**Table 1:** General characteristics of study population stratified for the MetS and non-MetS in 2009 (n = 2376).

Variable	MetS (n = 308)	Non-MetS (n = 2068)	*P*
Age (years)	42.59 ± 10.47	37.11 ± 10.99	0.000
BMI (kg/m^2^)	27.10 ± 3.10	22.76 ± 3.00	
WC (cm)	92.18 ± 7.22	79.16 ± 8.29	
SBP (mmHg)	131.78 ± 17.18	116.31 ± 14.13	
DBP (mmHg)	86.22 ± 11.49	75.67 ± 9.20	
TRIG (mmol/l)	2.50 (1.88,3.68)	1.03 (0.74,1.49)	
HDL (mmol/l)	1.28 ± 0.49	1.42 ± 0.30	
GLU (mmol/l)	6.13 ± 1.71	5.20 ± 0.75	
C3 (g/l)	1.25 ± 0.22	1.10 ± 0.22	0.000
C4 (g/l)	0.36 ± 0.09	0.33 ± 0.10	0.000
IgM (g/l)	1.39 ± 0.77	1.44 ± 0.77	0.148
IgA (g/l)	2.61 ± 1.06	2.47 ± 0.89	0.115
IgE (g/l)	128.65 (51.84, 290.75)	127.25 (52.01, 324.40)	0.699
IgG (g/l)	13.20 ± 2.77	13.43 ± 2.63	0.269
Smoking status, smokers, n (%)	174(56.5%)	1054(51%)	0.070
Alcohol consumption, drinkers, n (%)	144(46.8%)	813(39.3%)	0.013
Physical activity, physically active, n (%)	90(29.2%)	489(23.6%)	0.034
Family history of chronic disease, yes, n (%)	86(27.9%)	392(19.0%)	0.000

Data are presented as mean ± standard deviation (SD), and median (25 percentile, 75 percentile) or counts (percent).

Two-sided *t* test for categorical variables and Mann–Whitney U test for continuous variables.

MetS, metabolic syndrome; BMI, body mass index; SBP, systolic blood pressure; DBP, diastolic blood pressure; WC, waist circumference; TRIG, triglycerides; HDL, high-density lipoprotein cholesterol; GLU, glucose; C3, complement factor 3; C4, complement factor 4; IgM, immune globulin M; IgA, immune globulin A; IgE, immune globulin E; IgG, immune globulin G.

**Table 2:** Correlation coefficients and significance of association between serum C3 and C4 and metabolic variables after multi-factors adjustment.

Variable	C3		C4	
	*R*_1_	*P*_1_	*R*_2_	*P*_2_
BMI	0.436	0.000	0.221	0.000
WC	0.436	0.000	0.218	0.000
SBP	0.146	0.000	0.078	0.000
DBP	0.186	0.000	0.077	0.000
TRIG	0.128	0.000	0.029	0.153
HDL	−0.239	0.000	−0.146	0.000
GLU	0.108	0.000	0.039	0.057

Multi-factors: age, alcohol drinking status, smoking status, physical activity, and family history of chronic diseases.

Abbreviation: BMI, body mass index; WC, waist circumference; SBP, systolic blood pressure; DBP, diastolic blood pressure; TRIG, triglycerides; HDL, high density lipoprotein cholesterol; GLU, glucose; C3, complement factor 3; C4, complement factor 4.

**Table 3:** Odds ratios and 95% CI for MetS according to the C3 and C4 concentrations in 2009: a binary logistic regression.

		Total	MetS	Model 1 OR (95%CI)	*P*_1_	Model 2 OR (95%CI)	*P*_2_
C3	Q1 ( ≤ 0.967 g/l)	595	32	1.000		1.000	
	Q2 (0.968–1.104 g/l)	593	38	1.205 (0.742, 1.956)	0.452	1.413 (0.863, 2.313)	0.169
	Q3 (1.105–1.263 g/l)	596	83	2.847 (1.861, 4.354)	0.000	2.982 (1.934, 4.598)	0.000
	Q4 ( ≥ 1.264 g/l)	592	155	6.240 (4.181, 9.315)	0.000	7.047 (4.664, 10.648)	0.000
	*P*_*trend*_			0.000		0.000	
C4	Q1 ( ≤ 0.269 g/l)	598	48	1.000		1.000	
	Q2 (0.270–0.322 g/l)	596	65	1.403 (0.948, 2.075)	0.090	1.273 (0.855, 1.896)	0.235
	Q3 (0.323–0.382 g/l)	589	99	2.315 (1.606, 3.337)	0.000	2.017 (1.389, 2.928)	0.000
	Q4 ( ≥ 0.383 g/l)	593	96	2.213 (1.533, 3.195)	0.000	1.961 (1.349, 2.849)	0.000
	*P*_*trend*_			0.035		0.041	

Abbreviation: OR, odds ratio; CI, confidence interval; Q, quartile; C3, complement factor 3; C4, complement factor 4.

Model 1 was not adjusted.

Model 2 was adjusted for age, smoking status, alcohol drinking status, family history of chronic disease, and physical activity.

**Table 4:** RRs and 95% CI for MetS incidence in 2013 using the baseline levels of C3 and C4 from 2009: a Cox regression analysis.

		Total	MetS	Model 1	*P*_1_	Model 2	*P*_2_
				RR (95%CI)		RR (95%CI)	
C3	Q1 ( ≤ 0.955 g/l)	244	18	1.000		1.000	
	Q2 (0.966–1.103 g/l)	246	21	1.157 (0.617, 2.172)	0.649	1.249 (0.663, 2.354)	0.491
	Q3 (1.104–1.266 g/l)	242	45	2.521 (1.459, 4.354)	0.001	2.522 (1.459, 4.361)	0.001
	Q4 ( ≥ 1.267 g/l)	244	82	4.556 (2.735, 7.588)	0.000	4.779 (2.854, 8.003)	0.000
	*P*_*trend*_			0.000		0.000	
C4	Q1 ( ≤ 0.273 g/l)	246	21	1.000		1.000	
	Q2 (0.274–0.324 g/l)	243	37	1.784 (1.044, 3.047)	0.034	1.670 (0.975, 2.862)	0.062
	Q3 (0.325–0.385 g/l)	245	52	2.486 (1.498, 4.127)	0.000	2.309 (1.386, 3.845)	0.001
	Q4 ( ≥ 0.386 g/l)	242	56	2.711 (1.642, 4.476)	0.000	2.590 (1.567, 4.280)	0.000
	*P*_*trend*_			0.000		0.000	

Abbreviation: RR, relative risk; CI, confidence interval; Q, quartile; C3, complement factor 3; C4, complement factor 4.

C3 and C4 were respectively divided into 4 grades according to their interquartile range at baseline.

Model 1 was not adjusted.

Model 2 was adjusted for age, smoking status, alcohol drinking status, family history of chronic disease, and physical activity.

## References

[b1] International diabetes federation, *the IDF consensus worldwide definition of the metabolic syndrome*. (2006) Available at: http://www.idf.org/metabolic-syndrome. (accessed: 3rd April 2007)

[b2] Eckel R. H., Alberti K. G., Grundy S. M. & Zimmet P. Z. The metabolic syndrome. Lancet. 375, 181–183 (2010). 20109902 10.1016/S0140-6736(09)61794-3

[b3] Stern M. P. *et al.* Does the metabolic syndrome improve identification of individuals at risk of type 2 diabetes and/or cardiovascular disease? Diabetes care. 27, 2676–2681 (2004). 15505004 10.2337/diacare.27.11.2676

[b4] Ishino K., Mutoh M., Totsuka Y. & Nakagama H. Metabolic syndrome: a novel high-risk state for colorectal cancer. Cancer lett. 334, 56–61 (2013). 23085010 10.1016/j.canlet.2012.10.012

[b5] McNeill A. M. *et al.* The metabolic syndrome and 11-year risk of incident cardiovascular disease in the atherosclerosis risk in communities study. Diabetes care. 28, 385–390 (2005). 15677797 10.2337/diacare.28.2.385

[b6] Hotamisligil G. S. Inflammation and metabolic disorders. Nature. 444, 860–867 (2006). 17167474 10.1038/nature05485

[b7] Kovanen P. T., Manttari M., Palosuo T., Manninen V. & Aho K. Prediction of myocardial infarction in dyslipidemic men by elevated levels of immunoglobulin classes A, E, and G, but not M. Arch. Intern. Med. 158, 1434–1439 (1998). 9665352 10.1001/archinte.158.13.1434

[b8] Ridker P. M., Hennekens C. H., Buring J. E. & Rifai N. C-reactive protein and other markers of inflammation in the prediction of cardiovascular disease in women. New Engl. J. Med. 342, 836–843 (2000). 10733371 10.1056/NEJM200003233421202

[b9] Sesso H. D., Wang L., Buring J. E., Ridker P. M. & Gaziano J. M. Comparison of interleukin-6 and C-reactive protein for the risk of developing hypertension in women. Hypertension 49, 304–310 (2007). 17159088 10.1161/01.HYP.0000252664.24294.ff

[b10] Sesso H. D. *et al.* C-reactive protein and the risk of developing hypertension. Jama. 290, 2945–2951 (2003). 14665655 10.1001/jama.290.22.2945

[b11] Bertoni A. G. *et al.* Inflammation and the incidence of type 2 diabetes: the Multi-Ethnic Study of Atherosclerosis (MESA). Diabetes care. 33, 804–810 (2010). 20097779 10.2337/dc09-1679 PMC2845031

[b12] Bruno G. *et al.* C-reactive protein and 5-year survival in type 2 diabetes: the Casale Monferrato Study. Diabetes. 58, 926–933 (2009). 19074985 10.2337/db08-0900 PMC2661603

[b13] Duncan B. B. *et al.* Low-grade systemic inflammation and the development of type 2 diabetes: the atherosclerosis risk in communities study. Diabetes. 52, 1799–1805 (2003). 12829649 10.2337/diabetes.52.7.1799

[b14] Nilsson J., Jovinge S., Niemann A., Reneland R. & Lithell H. Relation between plasma tumor necrosis factor-alpha and insulin sensitivity in elderly men with non-insulin-dependent diabetes mellitus. Arterioscler. Thromb. Vasc. Biol. 18, 1199–1202 (1998). 9714125 10.1161/01.atv.18.8.1199

[b15] Jenny N. S. *et al.* Associations of inflammatory markers with coronary artery calcification: results from the Multi-Ethnic Study of Atherosclerosis. Atherosclerosis. 209, 226–229 (2010). 19766217 10.1016/j.atherosclerosis.2009.08.037 PMC2830357

[b16] Xu H. *et al.* Chronic inflammation in fat plays a crucial role in the development of obesity-related insulin resistance. J. Clin. Invest. 112, 1821–1830 (2003). 14679177 10.1172/JCI19451 PMC296998

[b17] Dandona P., Aljada A. & Bandyopadhyay A. Inflammation: the link between insulin resistance, obesity and diabetes. Trends. Immunol. 25, 4–7 (2004). 14698276 10.1016/j.it.2003.10.013

[b18] Hotamisligil G. S., Shargill N. S. & Spiegelman B. M. Adipose expression of tumor necrosis factor-alpha: direct role in obesity-linked insulin resistance. Science. 259, 87–91 (1993). 7678183 10.1126/science.7678183

[b19] Aso Y. *et al.* Metabolic syndrome accompanied by hypercholesterolemia is strongly associated with proinflammatory state and impairment of fibrinolysis in patients with type 2 diabetes: synergistic effects of plasminogen activator inhibitor-1 and thrombin-activatable fibrinolysis inhibitor. Diabetes care. 28, 2211–2216 (2005). 16123492 10.2337/diacare.28.9.2211

[b20] Zarkadis I. K., Mastellos D. & Lambris J. D. Phylogenetic aspects of the complement system. Dev. Comp. Immunol. 25, 745–762 (2001). 11602194 10.1016/s0145-305x(01)00034-9

[b21] Puchau B., Zulet M. A., Gonzalez de Echavarri A., Navarro-Blasco I. & Martinez J. A. Selenium intake reduces serum C3, an early marker of metabolic syndrome manifestations, in healthy young adults. Eur. J. Clin. Nutr. 63, 858–864 (2009). 18985060 10.1038/ejcn.2008.48

[b22] Phillips C. M. *et al.* Complement component 3 polymorphisms interact with polyunsaturated fatty acids to modulate risk of metabolic syndrome. Am. J. Clin. Nutr. 90, 1665–1673 (2009). 19828715 10.3945/ajcn.2009.28101

[b23] Onat A., Hergenc G., Can G., Kaya Z. & Yuksel H. Serum complement C3: a determinant of cardiometabolic risk, additive to the metabolic syndrome, in middle-aged population. Metabolism. 59, 628–634 (2010). 19913840 10.1016/j.metabol.2009.09.006

[b24] Grant R. W. & Dixit V. D. Adipose tissue as an immunological organ. Obesity (Silver Spring) 23, 512–518 (2015). 25612251 10.1002/oby.21003 PMC4340740

[b25] Gregor M. F. & Hotamisligil G. S. Inflammatory mechanisms in obesity. Annu. Rev. Immunol. 29, 415–445 (2011). 21219177 10.1146/annurev-immunol-031210-101322

[b26] van Oostrom A. J., Alipour A., Plokker T. W., Sniderman A. D. & Cabezas M. C. The metabolic syndrome in relation to complement component 3 and postprandial lipemia in patients from an outpatient lipid clinic and healthy volunteers. Atherosclerosis 190, 167–173 (2007). 16488421 10.1016/j.atherosclerosis.2006.01.009

[b27] Gabrielsson B. G. *et al.* High expression of complement components in omental adipose tissue in obese men. Obes. Res. 11, 699–708 (2003). 12805391 10.1038/oby.2003.100

[b28] Engstrom G., Hedblad B., Eriksson K. F., Janzon L. & Lindgarde F. Complement C3 is a risk factor for the development of diabetes: a population-based cohort study. Diabetes. 54, 570–575 (2005). 15677517 10.2337/diabetes.54.2.570

[b29] Nilsson B. *et al.* C3 and C4 are strongly related to adipose tissue variables and cardiovascular risk factors. Eur. J. Clin. Invest. 44, 587–596 (2014). 24754458 10.1111/eci.12275

[b30] Warnberg J. & Marcos A. Low-grade inflammation and the metabolic syndrome in children and adolescents. Curr. Opin. Lipidol. 19, 11–15 (2008). 18196981 10.1097/MOL.0b013e3282f4096b

[b31] Song K. *et al.* Serum immunoglobulin M concentration is positively related to metabolic syndrome in an adult population: Tianjin Chronic Low-Grade Systemic Inflammation and Health (TCLSIH) Cohort Study. PloS One 9, e88701 (2014). 24533139 10.1371/journal.pone.0088701 PMC3923043

[b32] Onat A. *et al.* Cross-sectional study of complement C3 as a coronary risk factor among men and women. Clin. Sci. (Lond) 108, 129–135 (2005). 15487975 10.1042/CS20040198

[b33] Phillips C. M. *et al.* Dietary fat, abdominal obesity and smoking modulate the relationship between plasma complement component 3 concentrations and metabolic syndrome risk. Atherosclerosis. 220, 513–519 (2012). 22138144 10.1016/j.atherosclerosis.2011.11.007

[b34] Wamba P. C. *et al.* Acylation stimulating protein but not complement C3 associates with metabolic syndrome components in Chinese children and adolescents. Eur. J. Endocrinol. 159, 781–790 (2008). 18805911 10.1530/EJE-08-0467

[b35] Cianflone K., Lu H., Smith J., Yu W. & Wang H. Adiponectin, acylation stimulating protein and complement C3 are altered in obesity in very young children. Clin. Endocrinol (Oxf) 62, 567–572 (2005). 15853826 10.1111/j.1365-2265.2005.02260.x

[b36] Ricklin D., Hajishengallis G., Yang K. & Lambris J. D. Complement: a key system for immune surveillance and homeostasis. Nat. Immunol. 11, 785–797 (2010). 20720586 10.1038/ni.1923 PMC2924908

[b37] Wlazlo N. *et al.* Complement factor 3 is associated with insulin resistance and with incident type 2 diabetes over a 7-year follow-up period: the CODAM Study. Diabetes care. 37, 1900–1909 (2014). 24760264 10.2337/dc13-2804

[b38] Phieler J. *et al.* The complement anaphylatoxin C5a receptor contributes to obese adipose tissue inflammation and insulin resistance. J. Immunol. 191, 4367–4374 (2013). 24043887 10.4049/jimmunol.1300038 PMC3817864

[b39] Lim J. *et al.* C5aR and C3aR antagonists each inhibit diet-induced obesity, metabolic dysfunction, and adipocyte and macrophage signaling. FASEB J. 27, 822–831 (2013). 23118029 10.1096/fj.12-220582

[b40] Phieler J., Garcia-Martin R., Lambris J. D. & Chavakis T. The role of the complement system in metabolic organs and metabolic diseases. Semin. Immunol. 25, 47–53 (2013). 23684628 10.1016/j.smim.2013.04.003 PMC3734549

[b41] Grundy S. M. *et al.* Diagnosis and management of the metabolic syndrome: an American Heart Association/National Heart, Lung, and Blood Institute scientific statement: Executive Summary. Crit. Pathw. Cardiol. 4, 198–203 (2005). 18340209 10.1097/00132577-200512000-00018

[b42] Ahren B., Havel P. J., Pacini G. & Cianflone K. Acylation stimulating protein stimulates insulin secretion. Int. J. Obes. Relat. Metab. Disord. 27, 1037–1043 (2003). 12917708 10.1038/sj.ijo.0802369

[b43] Zhou X. *et al.* Complement 3 activates the renal renin-angiotensin system by induction of epithelial-to-mesenchymal transition of the nephrotubulus in mice. Am. J. Physiol. Renal. Physiol. 305, F957–967 (2013). 23926185 10.1152/ajprenal.00344.2013

[b44] Zhang Y. *et al.* Endogenous sex hormones and C-reactive protein in healthy Chinese men. Clin. Endocrinol. (Oxf) 78, 60–66 (2013). 22313436 10.1111/j.1365-2265.2012.04359.x

[b45] Wu C. *et al.* The association of smoking and erectile dysfunction: results from the Fangchenggang Area Male Health and Examination Survey (FAMHES). J. Androl. 33, 59–65 (2012). 21436308 10.2164/jandrol.110.012542

[b46] Tan A. *et al.* Low serum osteocalcin level is a potential marker for metabolic syndrome: results from a Chinese male population survey. Metabolism. 60, 1186–1192 (2011). 21353261 10.1016/j.metabol.2011.01.002

