# Supplementary material for: Elevated serum complement factors 3 and 4 are strong inflammatory markers of the metabolic syndrome development: a longitudinal cohort study
Source: Sci Rep. 2016 Jan 4;6:18713. doi: 10.1038/srep18713 (PMC4698666; doi:10.1038/srep18713)
Supplement: Supplementary Information [file srep18713-s1.pdf]

Elevated serum complement factors 3 and 4 are strong inflammatory markers of the metabolic syndrome development: a longitudinal cohort study

Short running title: serum C3, C4 and metabolic syndrome.

Zhenfang Liu[1,2,3], Qin Tang[1], Jing Wen[1], Yan Tang[1], DaMin Huang[1,4], Yuzhen Huang[1], Jinling Xie[1], Yawen Luo[1], Min Liang[5], Chunlei Wu[1,6], Zheng Lu[1,7], Aihua Tan[1,8], Yong Gao[1], Qiuyan Wang[1,2,3], Yonghua Jiang[1,2,3] Ziting Yao[1,2,3], Xinggu Lin[1,2,3], Haiying Zhang[1,4], Zengnan Mo[1,2,3,7], Xiaobo Yang[1,4*]

***Corresponding author: Xiaobo Yang, E-mail: yxbo21021@163.com**

Supplemental table 1-Correlation coefficients of association between the IgE, IgG, IgM, IgA and other risk parameters of MetS after adjusting multi-factors.

Variable	IgE		IgG		IgM		IgA	
	R_1	P_1	R_2	P_2	R_3	P_3	R_4	P_4
BMI	-0.043	0.039	-0.043	0.035	-0.021	0.306	-0.019	0.360
WC	-0.050	0.014	-0.077	0.000	-0.042	0.040	-0.004	0.844
SBP	0.002	0.914	-0.007	0.748	-0.032	0.122	0.048	0.019
DBP	-0.030	0.139	-0.007	0.744	-0.032	0.120	0.053	0.010
TRIG	-0.029	0.155	-0.078	0.000	0.030	0.151	-0.019	0.359
HDL	0.035	0.087	-0.034	0.102	0.002	0.906	-0.039	0.060
GLU	0.011	0.580	0.001	0.975	0.003	0.895	0.067	0.001

Multi-factors: age, alcohol drinking status, smoking status, physical activity, family history of chronic diseases.

Abbreviation: BMI, body mass index; WC, waist circumference; SBP, systolic blood pressure; DBP, diastolic blood pressure; TRIG, triglycerides; HDL, high density lipoprotein cholesterol; GLU, glucose; IgM, immune globulin M; IgA, immune globulin A; IgE, immune globulin E; IgG, immune globulin G.

Supplemental table 2 -Odds ratios (ORs) and 95% CI for MetS according to the IgE, IgG, IgM and IgA concentrations: a binary logistic regression.

		Total	MetS	Model 1 OR(95%CI)	P_1	Model 2 OR(95%CI)	P_2
IgE	Q1 (≤52.045g/l)	594	77	1.000			
	Q2 (52.046-127.950 g/l)	594	76	0.985(0.701-1.383)	0.931	0.939(0.664-1.330)	0.724
	Q3 (127.951-318.850 g/l)	594	87	1.152(0.828-1.603)	0.401	1.086(0.774-1.525)	0.632
	Q4 (≥318.851 g/l)	594	68	0.868(0.613-1.229)	0.425	0.794(0.554-1.139)	0.210
IgG	Q1 (≤11.615 g/l)	594	91	1.000		1.000	
	Q2 (11.616-13.255 g/l)	594	70	0.738(0.528-1.032)	0.075	0.777(0.551-1.096)	0.151
	Q3 (13.256-14.940 g/l)	595	65	0.678(0.482-0.953)	0.025	0.760(0.535-1.079)	0.125
	Q4 (≥14.941 g/l)	593	82	0.887(0.642-1.225)	0.467	0.946(0.675-1.326)	0.746
IgM	Q1 (≤0.930 g/l)	606	84	1.000		1.000	
	Q2 (0.931-1.270 g/l)	586	83	1.025(0.739-1.422)	0.880	1.108(0.793-1.548)	0.549
	Q3 (1.271-1.760 g/l)	598	63	0.732(0.517-1.037)	0.079	0.807(0.566-1.152)	0.237
	Q4 (≥1.761 g/l)	586	78	0.954(0.685-1.329)	0.781	1.132(0.805-1.591)	0.477
IgA	Q1 (≤1.810 g/l)	600	71	1.000		1.000	
	Q2 (1.811-2.360 g/l)	594	79	1.143(0.811-1.610)	0.445	1.140(0.803-1.619)	0.462
	Q3 (2.361-2.980 g/l)	591	70	1.001(0.704-1.423)	0.995	1.027(0.716-1.474)	0.885
	Q4 (≥2.981 g/l)	591	88	1.304(0.932-1.823)	0.122	1.241(0.876-1.758)	0.225

Abbreviation: OR, odd ratios; CI, confidence interval; IgM, immune globulin M; IgA, immune globulin A; IgE, immune globulin E; IgG, immune globulin G.

Model 1 was not adjusted.

Model 2 was adjusted for age, smoking status, alcohol drinking status, family history of chronic disease, physical activity.
